# A Manual of the Practice of Medicine

**Published:** 1904-03

**Authors:** 


A Manual of the Practice of Medicine. By A. A. Stevens, A. M., M. D., Professor of Pathology in the Woman's Medical College of Pennsylvania; Lecturer on Physical Diagnosis in the University of Pennsylvania; Physician to the Episcopal Hospital and to St. Agnes' Hospital; Fellow of the College of Physicians of Philadelphia, etc. Sixth edition, thoroughly revised, enlarged and reset. Handsome post-octavo of 556 pages, illustrated. Philadelphia, New York, London: W. B. Saunders & Company. 1903. Flexible leather, $2.25 net.

This little work, now in its sixth edition, bound in flexible leather, its contents culled from the greatest medical thinkers of the country, is a handsome and valuable work. Much new material has been added in the more important ailments—malaria, gout, diseases of the digestive organs, blood, myocardium, cord, etc.

We take pleasure in recommending the work to students of medicine. J. M. L.

